# Extensive variation in chromosome number and genome size in sexual and parthenogenetic species of the jumping-bristletail genus *Machilis* (Archaeognatha)

**DOI:** 10.1002/ece3.1264

**Published:** 2014-10-07

**Authors:** Melitta Gassner, Thomas Dejaco, Peter Schönswetter, František Marec, Wolfgang Arthofer, Birgit C Schlick-Steiner, Florian M Steiner

**Affiliations:** 1 Institute of Ecology, University of InnsbruckTechnikerstraße 25, Innsbruck, 6020, Austria; 2 Institute of Botany, University of InnsbruckSternwartestraße 15, Innsbruck, 6020, Austria; 3 Institute of Entomology, Biology Centre ASCRBranisovska 31, Ceské Budejovice, 37005, Czech Republic

**Keywords:** Asexuality, chromosomal speciation, genome downsizing, parthenogenesis, polyploidy

## Abstract

Parthenogenesis in animals is often associated with polyploidy and restriction to extreme habitats or recently deglaciated areas. It has been hypothesized that benefits conferred by asexual reproduction and polyploidy are essential for colonizing these habitats. However, while evolutionary routes to parthenogenesis are manifold, study systems including polyploids are scarce in arthropods. The jumping-bristletail genus *Machilis* (Insecta: Archaeognatha) includes both sexual and parthenogenetic species, and recently, the occurrence of polyploidy has been postulated. Here, we applied flow cytometry, karyotyping, and mitochondrial DNA sequencing to three sexual and five putatively parthenogenetic Eastern-Alpine *Machilis* species to investigate whether (1) parthenogenesis originated once or multiply and (2) whether parthenogenesis is strictly associated with polyploidy. The mitochondrial phylogeny revealed that parthenogenesis evolved at least five times independently among Eastern-Alpine representatives of this genus. One parthenogenetic species was exclusively triploid, while a second consisted of both diploid and triploid populations. The three other parthenogenetic species and all sexual species were diploid. Our results thus indicate that polyploidy can co-occur with parthenogenesis, but that it was not mandatory for the emergence of parthenogenesis in *Machilis*. Overall, we found a weak negative correlation of monoploid genome size (Cx) and chromosome base number (x), and this connection is stronger among parthenogenetic species alone. Likewise, monoploid genome size decreased with elevation, and we therefore hypothesize that genome downsizing could have been crucial for the persistence of alpine *Machilis* species. Finally, we discuss the evolutionary consequences of intraspecific chromosomal rearrangements and the presence of B chromosomes. In doing so, we highlight the potential of Alpine *Machilis* species for research on chromosomal and genome-size alterations during speciation.

## Introduction

In animals, sexual reproduction prevails and asexuality is uncommon in most taxonomic groups (Suomaleinen et al. 1987). It is assumed that asexual organisms have originated from sexual ancestors (Bell 1982), and this transition can occur multiple times independently within a taxonomic group (Stenberg et al. 2003; Schwander and Crespi 2009; Bode et al. 2010; Elzinga et al. 2013). Apart from some entirely asexual clades (e.g., bdelloid rotifers or darwinulid ostracods, the so-called ancient asexuals; Judson and Normark 1996), strictly asexual animal species are rare (Bengtsson 2009; Vrijenhoek and Parker 2009). Instead, asexuality often occurs as alternative reproductive strategy within a sexual taxon. Vandel (1928) first used the term geographic parthenogenesis to describe the phenomenon of distinct geographic distribution of sexual and asexual populations within a species. Since then, many examples have been reported of parthenogenetic lineages being more common at higher altitudes, higher latitudes, and in anthropogenically disturbed or extreme habitats (e.g., recently deglaciated areas), compared with their sexual relatives. Following this, the advantage of asexuals over sexual congeners for colonizing new habitats has been attributed to their doubled reproductive potential (as predicted by the twofold cost of sex (Maynard Smith 1978)) and to highly specialized (“frozen niche variation model”) or generalized (“general purpose model”) genotypes (Vrijenhoek and Parker 2009). It has been unclear, although, to what extent asexuality itself (Cuellar 1977; Glesener and Tilman 1978; Law and Crespi 2002; Maniatsi et al. 2011) or frequent correlates like polyploidy (Zhang and Lefcort 1991; Stenberg et al. 2003; Comai 2005; Adolfsson et al. 2010) or hybridization (Kearney 2003, 2005; Ghiselli et al. 2007) promote the spread of organisms into these habitats. The proposed advantages of polyploidy and hybridization include protection against deleterious mutations (Comai 2005) and increased heterozygosity (Kearney 2005), respectively. However, in the latest review of empirical data (Lundmark and Saura 2006), polyploidy turned out to be the factor best explaining distributional patterns in species containing asexual populations.

Parthenogenesis is mostly associated with polyploidy in plants, and, to a lesser extent, this correlation holds for animals as well (Suomalainen 1940; Otto and Whitton 2000; Choleva and Janko 2013). The low occurrence of polyploid lineages in animals has been explained by dioecy (i.e., male and female gametes come from different individuals) and chromosomal sex determination (for a detailed discussion, see Otto and Whitton 2000 and references therein), but in fact, polyploidy may have been simply overlooked in many animal species (Mable 2004). Polyploidy in animals is thought to emerge more likely in parthenogenetic diploid populations, while in plants, polyploidy usually predates asexuality (Otto and Whitton 2000). Already Bell (1982) suggested that in animals, polyploidy may be fundamental for the persistence of parthenogenetic lineages but not essential for their emergence. Despite these theoretical predictions, empirical studies on the interactions between asexuality and polyploidy and their ultimate effect on the evolution and spatial distribution of species remain scarce.

To enhance our understanding of the interrelation between asexuality and polyploidy, it is thus fundamental to collect empirical data from previously neglected animal groups with high incidence of parthenogenesis. Jumping bristletails (Insecta: Archaeognatha) of the genus *Machilis* meet these criteria. Within the Eastern Alps, 25 nominal species are known and at least nine of them putatively reproduce via parthenogenesis, as only females have been reported (Wygodzinsky 1941; Janetschek 1954). Moreover, Dejaco et al. (2012) hypothesized the occurrence of polyploidy in a study including three *Machilis* species.

In this study, we apply relative genome-size measurements, karyotyping, and mitochondrial DNA sequencing to sexual and putative parthenogenetic *Machilis* species to address the following questions: (1) Did parthenogenesis arise once or multiple times independently across species? (2) Is parthenogenesis always coupled with polyploidy?

## Materials and Methods

### Specimen collection

We focused on eight species of which three reproduce sexually (*M. helleri*, *M. hrabei*, and *M. lehnhoferi*) and five putatively via parthenogenesis (*M. fuscistylis*, *M. pallida*, *M. engiadina*, *M. ticinensis*, and *M. tirolensis*). In the parthenogenetic species *M. ticinensis*, males have been reported from the southern Swiss Alps (Wygodzinsky 1941), potentially indicating a pattern of geographic parthenogenesis. In the absence of species-specific multilocus markers to prove asexual reproduction, we base our definition of parthenogenetic species on the following observations: (1) Four species, *M. engiadina*, *M. fuscistylis*, *M. pallida*, and *M. tirolensis*, have been collected numerously over the past 60 years but males were never found. (2) Between 2009 and 2013, we collected over 2000 specimens from these and other parthenogenetic *Machilis* species throughout the Eastern Alps. In doing so, we considerably widened the known distribution ranges of these four species (e.g., Rinnhofer et al. 2012; T. Dejaco, unpubl. data) but again did not find males. (3) In the sexual species we sampled, males occur at roughly even ratios and males do not differ from females in their habitat preferences. It is thus unlikely that we overlooked males in presumably parthenogenetic species. (4) The fifth parthenogenetic species included in this study, *M. ticinensis*, was originally described as sexual species from the southern Swiss Alps (Wygodzinsky 1941). Between 2009 and 2013, we repeatedly sampled female *M. ticinensis* on the northern side of the Alps (Austria and Switzerland), but never encountered male specimens. In contrast, male and female *M. ticinensis* were found at an even ratio in one sampling location in the southern Swiss Alps (MIR).

**Figure 1:**
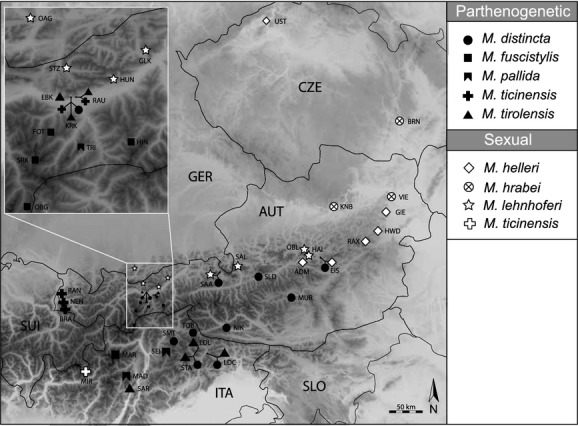
Geographic locations of all populations sampled in this study. White and black symbols correspond to sexual and parthenogenetic species, respectively. Whenever more than one species was found at a location, species-specific symbols are connected by lines with that location.

In total, 209 specimens were sampled at 41 geographically representative sites throughout the known distribution range of each species (Fig. 1; online supplementary material Table S1). Moreover, 12 specimens of four additional *Machilis* species were sampled: *M. glacialis*, *M. inermis*, *M. mesolcinensis*, and one unknown species, which morphologically keyed out as *M. glacialis*, but was named *M.* sp. A due to its distant position from *M. glacialis* in the mitochondrial phylogeny. These additional species were included in our phylogeny but excluded from other analyses due to poor geographic coverage. One specimen of *Lepismachilis y-signata* was used as out-group, resulting in a total of 222 specimens. Species were determined using the identification key in Palissa (1964) and original species descriptions (Riezler 1941; Wygodzinsky 1941; Kratochvíl 1945; Janetschek 1949). Sexing of individuals was based on secondary sexual organs – females are easily recognized by the presence of an ovipositor, detectable via screening a specimen's external morphology. In some cases, specimens were kept alive in a climate cabinet at 19°C until further use.

### DNA extraction, PCR conditions, and phylogenetic reconstruction

Genomic DNA was extracted from muscle tissue using the Mammalian Genomic DNA Miniprep Kit (Sigma-Aldrich, St. Louis, MO). Two newly designed primers, MachF5 (5′-TAGTTATACCYATYATAATYGGHGG-3′) and MachR7 (5′-CCTATRATAGCAAATACTGCYCC-3′), were used to amplify approx. 750 bp of the mitochondrial cytochrome c oxidase 1 gene (CO1). PCR conditions were as follows: 95°C for 2 min, 35 cycles (94°C for 30 sec, 50°C for 45 sec, 72°C for 90 sec), and 72°C for 10 min. Amplicons were checked via gel electrophoresis and purified using a mastermix containing the enzymes Exo1 (1 U/*μ*L) and FastAP (0.05 U/*μ*L) (Thermo Fisher Scientific Inc., Waltham, MA) and applying an incubation step at 37°C for 15 min, followed by denaturation of enzymes at 80°C for 15 min. Sanger sequencing was conducted by a commercial sequencing facility (Eurofins MWG Operon, Munich, Germany) using the forward primer MachF5. All sequences were deposited in GenBank (accession numbers KJ501697 – KJ501918). Sequences were checked for correct amino acid translation and aligned using the ClustalW algorithm implemented in MEGA 5 (Tamura et al. 2011), yielding a final alignment of 703 bp. The HKY+G model of nucleotide substitution was determined as best fitting our data based on the Akaike information criterion using jModeltest 2 (Guindon and Gascuel 2003; Darriba et al. 2012). Net between- and within-species genetic p-distances were calculated in MEGA 5. The Mann–Whitney *U*-test was used to test whether within-species p-distances differed significantly between sexual and parthenogenetic species.

A Bayesian inference tree was constructed with MrBayes 3.2 (Ronquist et al. 2012). Three partitions were specified corresponding to codon positions. Two parallel runs, each consisting of three heated and one cold chain, were run for 10^6^ generations, sampling trees every 1000 generations. Convergence was checked using the average standard deviation of splits frequencies, which fell below 0.01 after approx. 8.5 × 10^5^ generations. The first 500 trees were discarded as burn-in.

### Karyotyping

With minor adaptations, we followed the protocol of Sahara et al. (1999) for chromosome preparations. Specimens were anesthetized with carbon dioxide prior to dissection, and three legs were snap-frozen in liquid nitrogen and stored at −70°C for use in flow cytometry. Specimens were dissected in physiological solution containing 154 mmol/L NaCl, 5 mmol/L KCl, 1.8 mmol/L CaCl_2_, and 2.9 mmol/L NaHCO_3_. Gonads were removed and placed into hypotonic solution (0.075 mol/L KCl) for swelling (15–20 min), followed by fixation in Carnoy's solution (ethanol: chloroform: acetic acid = 6:3:1) for 20 to 30 min. Immediately after the removal of gonads, specimens were preserved in 99% ethanol and stored at −20°C for further use. Glass slides were incubated in acid ethanol (1% HCl: 96% ethanol = 1:100) for at least half an hour before use. Gonads were dispersed on dry-cleaned slides in a drop of acetic acid (60%) with tungsten needles and spread using a heating plate (40°C).

Chromosome preparations were first checked for metaphase spreads under a Nikon Eclipse E600 phase contrast microscope and then stained with 0.02 *μ*g/mL DAPI in antifade solution based on DABCO (1,4-diazabicyclo[2.2.2]octane; Sigma-Aldrich). Pictures of chromosome spreads were taken with a Leica DFC 495 digital camera (Leica Microsystems, Wetzlar, Germany) mounted on a Leica DM 5000B fluorescence microscope. Single chromosomes were arranged according to their size using Adobe Creative Suite 4 software (Adobe Systems, San Jose, CA). Chromosome number was counted in at least three metaphase spreads per individual. Chromosome base number (x; i.e., the haploid number, which is occurring twice in a diploid and three times in a triploid) was calculated by dividing diploid chromosome number by two and triploid chromosome numbers by 3. Fundamental number of arms (FN; i.e., the total number of chromosome arms in a 2n metaphase complement) was determined for all species from representative karyotypes of as many sampling locations as possible.

In *M. ticinensis*, chromosome lengths (in *μ*m) were measured in each one karyotype of nine asexual and five sexual individuals using the software MicroMeasure version 3.3 (Reeves 2000). We calculated mean chromosome length (MChL, equal to the sum of chromosome lengths divided by the number of chromosomes within one karyotype) and tested for significant difference in MChL among sexual and asexual individuals. Statistical tests were calculated in SigmaPlot v.12.5 (Systat Software Inc., San Jose, CA), applying a significance level of *α* = 0.05. When normality and equal variances were given, we applied parametric tests. Whenever one of these criteria failed, we used nonparametric tests instead (Reeves 2000).

### Flow cytometry measurements

We applied flow cytometry to obtain relative genome-size estimates from leg muscle tissue using *Bellis perennis* (Asteraceae; 2C = 3.38 pg, Schönswetter et al. 2007) as internal standard (i.e., measured together with each sample). Samples were treated following Suda et al. (2007). In detail, single legs and approx. 0.5 cm^2^ of a *B. perennis* leaf were chopped with 500 *μ*L of ice cold Otto 1 Buffer (0.1 mol/L citric acid, 0.5% Tween20 (Merck KGaA, Darmstadt, Germany)) in a small Petri dish. The suspension was then filtered through a 42-*μ*m nylon mesh and incubated for a few minutes. Finally, 1 mL of staining solution (DAPI (4 *μ*L/mL) and 2-mercaptoethanol (2 *μ*L/mL) in Otto 2 buffer (0.4 mol/L Na_2_HPO_4_∙12 H_2_O)) was added. Fluorescence intensity of 3000 cell nuclei per sample was measured using a Partec CyFlow® space flow cytometer (Partec GmbH, Münster, Germany). Gating and peak analysis were performed automatically using the Partec Flo Max software. In a few samples that were defrosted too fast, fluorescence peaks showed increased noise on the left side. In these cases, left gates were adjusted manually to prevent overestimation of peak width.

Genome size measurements based on DAPI fluorescence have been criticized because of DAPI's AT-specific DNA-binding properties. Potential differences in genomic AT/GC content among species may introduce some bias to fluorescence intensity and thus genome size estimates. However, Johnston et al. (1999) showed that genome size estimates based on DAPI largely agree with estimates based on other fluorochromes. As we used a plant (*B. perennis*) as internal standard, we report relative genome size (i.e., fluorescence intensity of sample divided by fluorescence intensity of internal standard), instead of calculating nuclear DNA content in picograms. Relative genome size values were divided by ploidy level to obtain monoploid genome size (Cx), which is comparable among different ploidy levels. Fig. 4 was drawn in Sigmaplot v.12.5. Fig. 5 was generated using the R package ggplot2 (http://cran.r-project.org/web/packages/ggplot2/index.html).

## Results

For each species, an overview of sampling locations is given in Table 1, including number of female and male individuals sampled, mean chromosome number and relative genome size, ploidy level, elevation, and FN.

**Table 1:** Summary of average chromosome number and genome size (mean ± SD) values for all *Machilis* populations sampled in this study, including additional information on reproductive mode, number of individuals per population, and sex

Species name	Repr. mode	Sampling location	Number of specimens		Number of chromosomes		FN	Mean relative genome size (±SD)		Ploidy level
		
			F	M	F (2n)	M (n)		F	M	
*Machilis helleri*	S	ADM (AT)	2	2	52	26	124	1.49 ± 0.01	1.46 ± 0.02	2n
	S	EIS (AT)	4	1	52	26	124	1.56 ± 0.3	1.45	2n
	S	GIE (AT)	5	4	n/a	25, 51^1^	n/a	1.60 ± 0.01	1.60 ± 0.01	2n
	S	HWD (AT)	8	3	50	25	124	1.60 ± 0.02	1.57 ± 0.01	2n
	S	RAX (AT)	3	1	n/a	25	n/a	1.55 ± 0.04	1.49	2n
	S	UST (CZ)	4	0	52	n/a	n/a	1.45 ± 0.02	n/a	2n
*Machilis hrabei*	S	BRN (CZ)	4	6	52	26	140	1.90 ± 0.02	1.89 ± 0.02	2n
	S	KNB (AT)	3	5	52	26	140	1.83 ± 0.01	1.77 ± 0.02	2n
	S	VIE (AT)	4	2	52	26	140	1.86 ± 0.02	1.85 ± 0.03	2n
*Machilis lehnhoferi*	S	GLK (AT)	4	1	52	26, 52^1^	128	1.73 ± 0.3	1.77	2n
	S	HAI (AT)	3	0	52	n/a	128	5.82 ± 0.03	n/a	2n
	S	HUN (AT)	1	1	52	26, 52^1^	n/a	1.7	1.77	2n
	S	OBE (DE)	3	2	52	26	n/a	1.76 ± 0.02	1.76 ± 0.08	2n
	S	OBL (AT)	4	0	52	n/a	128	1.71 ± 0.05	n/a	2n
	S	SAA (AT)	0	3	n/a	26	n/a	n/a	1.77 ± 0.01	2n
	S	SAL (AT)	0	4	n/a	26	n/a	n/a	1.76 ± 0.02	2n
	S	STZ (AT)	3	0	52	n/a	n/a	1.72 ± 0.02	n/a	2n
*Machilis distincta*	P	EIS (AT)	10		50		140	1.73 ± 0.03		2n
	P	KRK (AT)	1		50		n/a	1.73		2n
	P	LDC (IT)	1		50		n/a	1.74		2n
	P	MUR (AT)	9		50		n/a	1.73 ± 0.01		2n
	P	NIK (AT)	8		50		n/a	1.72 ± 0.02		2n
	P	SAA (AT)	2		50		n/a	1.74 ± 0.00		2n
	P	SLD (AT)	4		50		140	1.74 ± 0.02		2n
	P	SMT (IT)	4		50		140	1.72 ± 0.02		2n
	P	STA (IT)	2		50		n/a	1.73 ± 0.03		2n
	P	TOB (IT)	1		50		n/a	1.73		2n
*Machilis fuscistylis*	P	FOT (AT)	3		54		148	1.46 ± 0.02		2n
	P	HIN (AT)	6		56		156	1.50 ± 0.01		2n
	P	MAR (IT)	1		54		148	1.47		2n
	P	OBG (AT)	4		54		148	1.47 ± 0.03		2n
	P	SRK (AT)	1		54		n/a	1.46		2n
*Machilis pallida*	P	MAD (IT)	8		78		n/a	2.30 ± 0.03		3x
	P	SEI (IT)	4		78		186	2.29 ± 0.02		3x
	P	TRI (AT)	5		78		n/a	2.31 ± 0.03		3x
*Machilis tirolensis*	P	EBK (AT)	4		75		198	2.54 ± 0.03		3x
	P	KRK (AT)	2		75		n/a	2.54 ± 0.04		3x
	P	LDC (IT)	6		50		132	1.61 ± 0.02		2n
	P	LDL (IT)	3		50		132	1.62 ± 0.02		2n
	P	RAU (AT)	1		75		n/a	2.54		3x
	P	SAR (IT)	3		75		n/a	2.54 ± 0.02		3x
	P	STA (IT)	3		50		n/a	1.60 ± 0.01		2n
*Machilis ticinensis*	P	BRA (AT)	2		46		128	1.67 ± 0.06		2n
	P	KRK (AT)	1		46		n/a	1.65		2n
	P	NEN (AT)	7		46		128	1.69 ± 0.01		2n
	P	RAN (AT)	1		46		n/a	1.67		2n
	P	RAU (AT)	2		46		128	1.68 ± 0.02		2n
	S	MIR (CH)	8	2	46, 46 + Bs	23	128	2.01 ± 0.06	1.95 ± 0.01	2n
Total		41	172	37						

FN, fundamental number of arms; F, females; M, males; S, sexual; P, parthenogenetic; n/a, not available (or not applicable); AT, Austria; CZ, Czech Republic; DE, Germany; IT, Italy; CH, Switzerland; Bs, B chromosomes.

1 2n chromosome numbers are additionally given in males when mitotic chromosomes were found.

### Mitochondrial phylogeny

**Figure 2:**
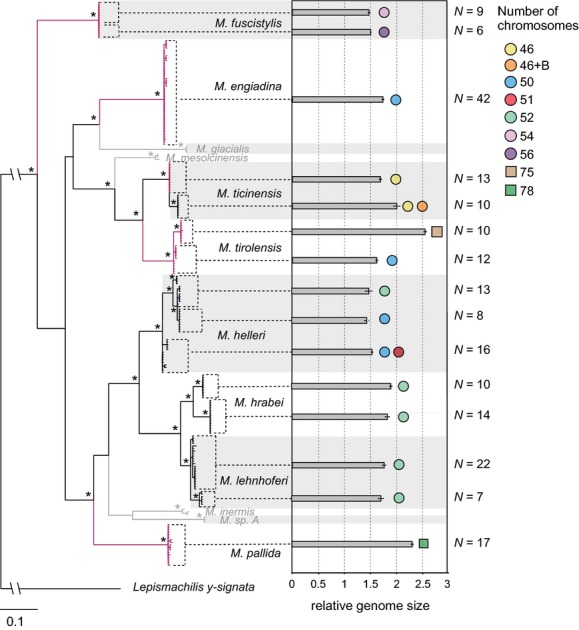
Bayesian phylogeny based on 222 mitochondrial CO1 sequences from 12 Eastern-Alpine *Machilis* species and *Lepismachilis y-signata* as out-group. All nodes supported by posterior probabilities higher than 0.95 are indicated by stars. Red branches highlight parthenogenetic species. Species included in the phylogeny but excluded from other analyses have gray branches. Mean relative genome size and chromosome number are given for all species and major intraspecific clades. Diploid and triploid chromosome numbers are indicated by circled and squared symbols, respectively. The number of individuals used to calculate average genome size values (N) is given to the right side of bars. Error bars indicate standard deviation (SD).

Our phylogeny included eight target species, as well as four additional species mentioned in the specimen collection section. All species formed monophyletic clusters supported by posterior probabilities above 0.95 (Fig. 2). In contrast, some deeper nodes were only weakly supported. The three sexual species formed a monophyletic group, while the parthenogenetic species did not. Mean net p-distances between species ranged between 3.00% and 16.22%, while p-distances within-species were equal or lower than 1.00% (Table 2). Mean within-group distances were significantly higher in sexual than in parthenogenetic species (Student's *t*-test: *t* = −3.369; df = 6; *P* = 0.015).

**Table 2:** Genetic *P*-distances (in %) within (±SD) and among species. Rows 1–3 are sexual species, rows 4–8 (light gray background) are parthenogenetic species, and rows 9–12 (dark gray background) are species that were included in the phylogeny but not in other analyses due to low sample size

S. no	Species	Mean within group distance	Pairwise mean net between distance\standard deviation											

			1	2	3	4	5	6	7	8	9	10	11	12
1	*Machilis helleri*	0.9 (±0.2)		1.03	1.04	1.37	1.45	1.45	1.36	1.32	1.61	1.18	1.35	1.29
2	*Machilis hrabei*	1.0 (±0.3)	7.61		0.57	1.51	1.49	1.48	1.44	1.48	1.49	1.37	1.53	1.36
3	*Machilis lehnhoferi*	0.6 (±0.2)	7.38	3.04		1.48	1.43	1.55	1.35	1.47	1.63	1.34	1.53	1.44
4	*Machilis ticinensis*	0.5 (±0.2)	12.48	14.56	13.78		1.49	1.50	1.59	0.89	1.53	1.47	1.13	1.51
5	*Machilis distincta*	0.1 (±0.0)	13.55	13.97	13.46	14.52		1.52	1.33	1.49	1.40	1.51	1.48	1.66
6	*Machilis fuscistylis*	0.0 (±0.0)	13.51	14.31	14.81	13.99	14.60		1.55	1.55	1.57	1.48	1.45	1.51
7	*Machilis pallida*	0.1 (±0.1)	11.96	12.99	12.06	15.93	12.75	15.00		1.55	1.59	1.42	1.53	1.49
8	*Machilis tirolensis*	0.4 (±0.2)	12.49	14.44	14.17	5.95	14.77	15.10	15.20		1.52	1.41	1.16	1.48
9	*Machilis glacialis*	0.0 (±0.0)	15.67	14.91	15.93	14.38	13.60	15.22	15.40	13.80		1.49	1.59	1.62
10	*Machilis inermis*	0.5 (±0.2)	10.15	12.75	12.25	13.54	14.20	14.08	12.65	12.69	14.27		1.40	1.32
11	*Machilis mesolcinensis*	0.3 (±0.2)	12.23	14.59	14.53	9.07	14.39	13.70	14.63	9.37	14.94	13.37		1.50
12	*Machilis spA*	0.0 (±0.0)	11.78	12.14	12.56	14.68	16.15	14.65	13.98	14.51	16.22	11.57	15.08	

**Figure 3:**
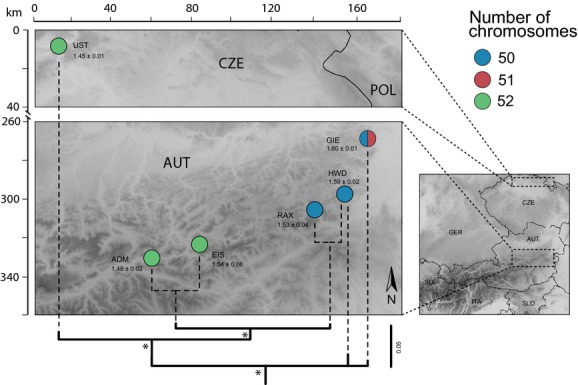
Geographic locations of sampled *Machilis helleri* populations with corresponding number of chromosomes and mean relative genome size (±SD). Below, a simplified drawing of the corresponding branch of the mitochondrial phylogeny is displayed. All nodes supported by posterior probabilities higher than 0.95 are indicated by stars.

Intraspecific splits occurred in *M. helleri*, *M. hrabei, M. ticinensis,* and *M. tirolensis* (Fig. 2). In *M. ticinensis* and *M. tirolensis*, these splits were congruent with reproductive mode and ploidy level, respectively. In *M. helleri*, the two resulting clades were partly congruent with differing chromosome number (Fig. 2 and Fig. 3). In *M. hrabei*, the two clades corresponded to sampling locations from Austria (KNB, VIE) and the Czech Republic (BRN; see Table 1).

### Chromosome numbers

We found predominantly mitotic chromosomes in female gonads and meiotic chromosomes in male gonads, except in two male specimens (each one *M. helleri* and *M. lehnhoferi*), where both meiotic and mitotic chromosomes were found. In most sampling locations of the three sexual species (*M. helleri*, *M. hrabei,* and *M. lehnhoferi*), we consistently counted 2n = 52 chromosomes in females and *n* = 26 bivalents in males. The assembled karyotypes of these species revealed that they differed in the numbers of metacentric, submetacentric, and acrocentric chromosomes, and hence also in the fundamental number of arms (FN; Table 1, Fig. S1). In three locations of *M. helleri*, however, we found only 2n = 50 in females and *n* = 25 bivalents in males (HWD and RAX), and 2n = 51 chromosomes and *n* = 25 bivalents in one male from GIE (Fig. 3; Table 1). Unfortunately, no unambiguously countable chromosome spread could be produced in female *M. helleri* specimens from GIE and RAX. All *M. helleri* specimens with 52 chromosomes fell into one well-supported mitochondrial subclade, including some specimens from locations RAX (all) and HWD (six individuals), which had only 50 chromosomes. The remaining individuals from HWD (five individuals) and GIE corresponded to the basal subclade within *M. heller*i (Fig. 3), having 50 chromosomes.

In asexual species, chromosome number ranged from 2n = 46 to 2n = 3x = 78 (Fig. 2, Table 1). In *M. fuscistylis*, we detected intraspecific variation with 2n = 54 chromosomes across four locations (OBG, FOT, MAR, and SRK) but consistently 2n = 56 chromosomes in specimens from HIN (Fig. 2 and Fig. S1). In all *M. pallida* specimens, we counted 2n = 3x = 78 chromosomes. In *M. tirolensis*, most sampling locations harbored exclusively diploid specimens (2n = 50 chromosomes), but at two locations (LDL and LDC), only triploids with 2n = 3x = 75 chromosomes were found (Fig. S1). We consistently found 2n = 50 chromosomes in specimens of *M. engiadina*.

In *M. ticinensis*, both males and females were found at location MIR, while exclusively females were found at the other five sampling locations (see Table 1), possibly indicating a pattern of geographic parthenogenesis. In the sexual population, specimens had 2n = 46 chromosomes plus a varying number of supernumerary elements, that is, B chromosomes, representing 2n = 46 (*N* = 3), 2n = 46 + 1B (*N* = 1), 2n = 46 + 4B (*N* = 3), and 2n = 46 + 5B (*N* = 1) in females, and *n* = 23 (*N* = 2) bivalents in male meiotic spreads (online supplementary material Fig. S1 and Tab. S1). In contrast, parthenogenetic specimens consistently had 2n = 46 chromosomes and no B chromosomes. In specimens from the sexual population, mean chromosome size was significantly larger compared with parthenogenetic specimens (Mann–Whitney *U*-test; U = 0, *P* = 0.003, N_sex_ = 5, 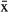
_sex_ = 4.26 ± 0.38 *μ*m, N_parth_ = 9, 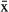
_parth_ = 3.33 ± 0.12 *μ*m).

**Figure 4:**
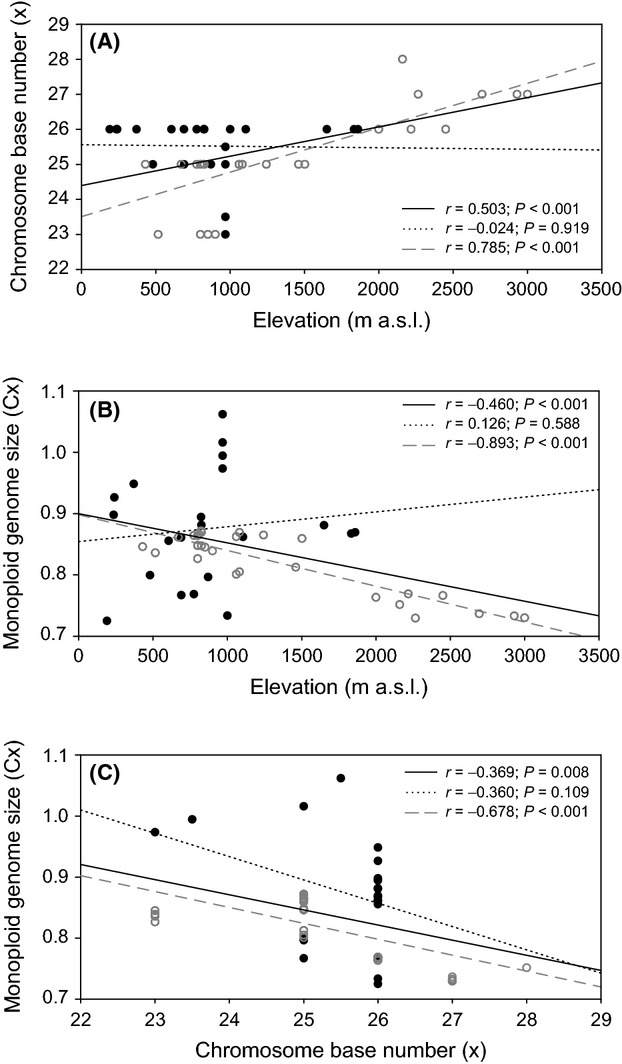
Correlation between elevation and chromosome base number (A), elevation and monoploid genome size (B), and chromosome base number and monoploid genome size (C). Sexual populations are represented by black dots, parthenogenetic populations by gray circles. Regression lines and correlation coefficients including *P*-values are given for all populations together (black line), just sexual populations (black dotted line), and just parthenogenetic populations (gray dashed line).

Chromosome base numbers (x) significantly increased with elevation across all species (Pearson's *r*: *r* = 0.503, *P* < 0.001; Fig. 4A). However, sexual species did not contribute to this trend as no correlation was found among populations of sexual species alone (Pearson's *r*: *r* = −0.024, *P* = 0.919; Fig. 4A). In contrast, when only populations from parthenogenetic species were considered, the correlation was strongest (Pearson's r: *r* = 0.785, *P* < 0.001; Fig. 4A). No such correlation was found within any of the single species (data not shown).

### Relative genome size measurements

**Figure 5:**
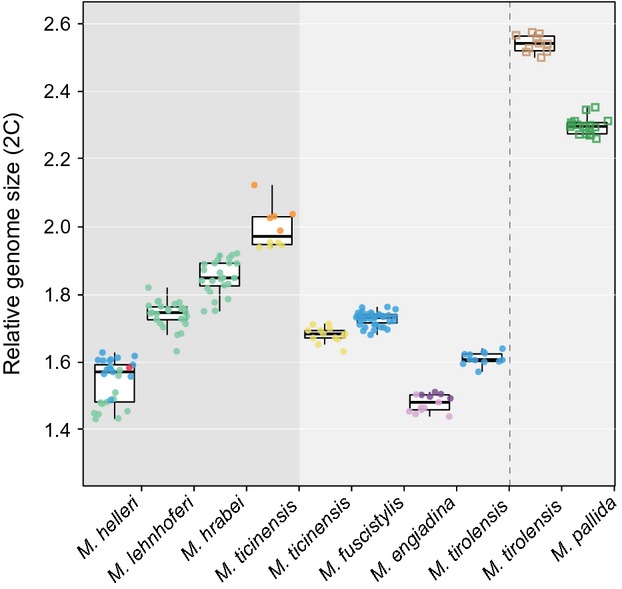
Relative genome size plotted for 191 *Machilis* individuals for which chromosome number has been determined as well. Individual measurements are grouped according to species affiliation and, in *M. ticinensis* and *M. tirolensis*, split according to reproductive mode (sexual/parthenogenetic) and ploidy level (diploid/triploid), respectively. Dark and light gray backgrounds indicate sexual and parthenogenetic mode of reproduction, respectively. Diploid individuals are represented by filled circles and triploids by empty squares. Chromosome number is coded using the same colors as in Fig. 2. Within groups, random horizontal scatter was applied to individuals for better visualization.

The relative genome sizes (2C) ranged from 1.43 (*M. helleri*) to 2.12 (*M. ticinensis*) in diploids and from 2.26 (*M. pallida*) to 2.57 (*M. tirolensis*) in triploids (Fig. 2 and Fig. 5). This approximately corresponds to C values ranging from 2.21 pg to 3.59 pg in diploids and 3.82 pg to 4.35 pg in triploids. Even when taking into account potential bias introduced by DAPI staining and using a plant as an internal standard, these values are higher than mean C values reported for most insect orders (except Orthopthera), and a similarly high C value has been reported for the closely related insect order Zygentoma (*Thermobia domestica*: C = 3.09 pg; Gregory 2014). Among sexual species, relative genome size (2C) varied significantly in overall (Kruskal–Wallis ANOVA: *H* = 66.388, df = 2, *P* < 0.001) and multiple pairwise comparisons (Dunn's test: *M*. *hrabei* vs. *helleri*: *Q* = 8.086, *P* < 0.05; *M*. *hrabei* vs. *lehnhoferi*: *Q* = 3.466, *P* < 0.05; *M*. *lehnhoferi* vs. *helleri*: *Q* = 4.629, *P* < 0.05). In *M. hrabei*, 2C values varied significantly among the two mitochondrial subclades (Student's *t*-test: *t* = −5.33, df = 22, *P* < 0.001), with specimens from the Czech Republic (BRN) having larger genomes than specimens from Austria. Intraspecific variation (as measured by standard deviations of relative genome size within each species) was significantly higher in sexual than in parthenogenetic species when *M. ticinensis* was excluded (Student's *t*-test: *t* = −2.448, df = 5, *P*_one-tailed_ = 0.029; see Fig. 5).

In the parthenogenetic species *M. fuscistylis*, specimens with 56 chromosomes had significantly larger relative genome sizes than specimens with 54 chromosomes (Student's *t*-test: *t* = −3.919, df = 11, *P* = 0.002). In triploid individuals of *M. tirolensis*, relative genome size was 1.58-fold compared with conspecific diploids, thus corroborating their triploid status. In the potentially geographic parthenogenetic species *M. ticinensis*, relative genome size was significantly larger in the sexual population compared with parthenogenetic specimens (Mann–Whitney *U*-test: *U* = 0, N_parth._ = 13, N_sex_ = 10, *P* < 0.001). Within the sexual *M. ticinensis* population, relative genome size increased significantly with the number of B chromosomes (Pearson's *r*: *r* = 0.939, *P* < 0.001). Nevertheless, relative genome size of sexual *M. ticinensis* specimens without B chromosomes (2n = 46) was significantly larger than relative genome size in parthenogenetic specimens (Student's *t*-test: *t* = −25.211, df = 16, *P* < 0.001; Fig. 5).

Monoploid genome sizes (Cx) decreased significantly with increasing elevation across all species (Pearson's r: *r* = −0.460, *P* < 0.001; Fig. 4B). Sexual species did not contribute to this trend, as no correlation was found among populations from sexual species alone (Pearson's r: *r* = 0.126, *P* = 0.588; Fig. 4B). Among parthenogenetic species, however, the negative correlation of Cx and elevation was strongest (Pearson's r: *r* = −0.893, *P* < 0.001; Fig. 4B). Similarly, a considerable negative correlation of Cx and chromosome base number (x) was found among parthenogenetic (Pearson's r: *r* = −0.678, *P* < 0.001; Fig. 4C) but not among sexual species (Pearson's r: *r* = −0.360, *P* = 0.109; Fig. 4C). When populations from all species were considered, this correlation was very weak (Pearson's r: *r* = −0.369, *P* < 0.001; Fig. 4C).

## Discussion

In this study, we investigated the link between parthenogenesis and polyploidy in Alpine representatives of the jumping-bristletail genus *Machilis* by generating relative genome-size estimates and karyotypes from geographically representative samples of three sexual and five presumably parthenogenetic species. By mapping relative genome size and chromosome numbers onto a mitochondrial phylogeny, we tackled the questions raised in the introduction and identified promising trajectories for future research.

### Multiple origin of parthenogenesis

As this is the first phylogenetic framework in the genus *Machilis*, no ‘a priori’ hypotheses concerning species relationships and evolution of parthenogenesis were available. Assuming that a reversal from parthenogenetic to sexual reproduction is unlikely following Dollo's law (Gould 1970), the nonmonophyly of parthenogenetic species indicated that asexuality originated at least five times independently (see Fig. 2). Multiple evolution of parthenogenesis within a genus has been demonstrated in other animal groups such as stick insects (Schwander and Crespi 2009) and lizards (Manriquez-Moran et al. 2014), and these groups offer exciting opportunities for studying evolutionary scenarios leading to parthenogenesis.

In Alpine *Machilis* species, multiple origins of parthenogenesis may have been facilitated by Pleistocene glaciation cycles, which are thought to have increased the incidence of asexual reproduction in plants and animals (Hörandl 2009). In fact, persistence on so-called nunataks (i.e., rocky peaks towering above the ice shield) has been suggested in two alpine *Machilis* species (*M. fuscistylis*: Janetschek 1956; *M. pallida*: Wachter et al. 2012) and may be linked to the onset of parthenogenesis in these species. In contrast, inner-Alpine persistence of glaciations at lower elevations is unlikely. In species occurring at lower elevations (e.g., *M. engiadina*, *M. ticinensis*, and *M. tirolensis*), parthenogenesis is thus more likely to have evolved during recolonization of the inner-Alpine area following persistence in peripheral refugia. However, in the absence of a robust time calibration, dating speciation events and origins of parthenogenesis remain elusive. In line with the concept of geographic parthenogenesis (Vandel 1928; Hörandl 2009), parthenogenetic species included in our study mainly occurred in central parts of the Alps, whereas sexual species were mostly scattered close to the margin of the Alps and in surrounding lowland areas.

### Incidence of polyploidy

Polyploidy was found in two parthenogenetic species. In *M. tirolensis,* populations with both, 50 and 75 chromosomes were identified. Because relative genome size in the latter was on average 1.58 times higher than in the former, the latter's triploid status is strongly supported. The second case of triploidy is hypothesized in *M. pallida* (2n = 3x = 78) based on the 1.5 ratio in chromosome number compared with sexual species. However, the direct sexual ancestor of *M. pallida* is unknown and possibly extinct, and thus, accurate inference of a phylogenetically unbiased genome size ratio is not possible. Still, a ratio of 1.34 was calculated using the average relative genome size across sexual species included in this study. Deviation from the expected 1.5 ratio might be explained by phylogenetic distance between *M. pallida* and these sexual taxa.

Since the other three parthenogenetic species were diploid, polyploidy is not always coupled with parthenogenesis in *Machilis*. Similarly, parthenogenetic diploids have been found in numerous other arthropod taxa, for example, brine shrimps (Maccari et al. 2013), weevils (Stenberg et al. 2003), and stick insects (Schwander and Crespi 2009). Our results are thus in line with the hypothesis, that in animals, polyploidy emerges secondarily, after the establishment of diploid parthenogenetic populations (Suomaleinen et al. 1987).

### Opportunities for studying genome evolution in the genus Machilis

In this study, we found substantial variation in chromosome number and relative genome size among and within *Machilis* species. In the following, we highlight three peculiarities in our data that emphasize the potential of the genus *Machilis* as a study system for investigating the role of chromosomal rearrangements and genome size alterations in evolutionary diversification.

#### Genome downsizing

Monoploid genome size (Cx) was negatively correlated with chromosome base number (x) among species included in this study, and this correlation was strongest among parthenogenetic species. Moreover, while Cx decreased with elevation, chromosome base number tended to increase with elevation, and this effect was also stronger among parthenogenetic species. We cautiously interpret this as a signal of genome downsizing along an altitudinal gradient that is possibly stronger in parthenogenetic than in sexual species. Downsizing of nuclear DNA content is a known phenomenon in polyploid plants, where higher ploidy levels tend to have smaller C values than diploids of a given species (Leitch and Bennett 2004). Here, in contrast, we hypothesize that genome size and chromosome number might be shaped by environmental conditions and that parthenogenetic *Machilis* species might be more prone to this process than sexual congeners.

To date, specific mechanisms involved in genome size reductions are largely unclear. However, Hessen et al. (2010) proposed that reductions in genome size in eukaryotes may be explained by the need for streamlined genomes in environments selecting for rapid growth due to low food availability. As in alpine environments (i.e., above 2000 m a.s.l.) activity periods are short and food availability is low, we suggest that harsh climatic conditions selected for smaller genomes in alpine *Machilis* species. Alternatively, our interpretation might be misled by the fact that no exclusively alpine sexual species are included in our study. One reason for this is the unclear taxonomic situation in the genus *Machilis* (Dejaco et al. 2012), with several species being poorly delimited and thus not available for evolutionary research. Also, most putative *Machilis* species found in alpine habitats are presumably parthenogenetic, and one could expect that asexual reproduction itself is a crucial property enabling persistence in this habitat. Our results uncover a potential alternative hypothesis by pointing at smaller genomes (and more and thus smaller chromosomes) in alpine species. Future work should aim at validating this correlation in sexual alpine *Machilis* species to disentangle the relative contribution of genome size reduction versus asexual reproduction to the persistence in alpine environments.

#### Intraspecific chromosomal rearrangements

We found intraspecific variation in chromosome number within two species. In the parthenogenetic *M. fuscistylis,* one population (HIN) harbored two additional chromosomes compared with the other populations. As the fundamental number of arms differed between these subgroups, we excluded the possibility of chromosome fusion or fission, as both types of rearrangement would not alter FN. This assumption is further supported by relative genome size measurements, which confirmed a significantly higher 2C value in the HIN population. We propose that the duplication of one chromosome pair in HIN (Fig. S1) is more parsimonious than the parallel loss of one chromosome pair in the other populations. Also, as both karyotypic groups shared the same CO1 haplotype, the novel karyotype must have originated recently. We stress the importance of chromosomal rearrangements and karyotypic variation as a potential source of divergence among parthenogenetic lineages, which is in line with postulates by others (Sunnucks et al. 1998; Blackman et al. 2000).

Differing chromosome numbers in the sexual species *M. helleri* (52 vs. 50 chromosomes, one male with 51) showed that chromosomal rearrangements are not restricted to parthenogenetic *Machilis* species. As the fundamental number of arms was congruent across different karyotypes, the change in chromosome number is best explained by fission of one or fusion of two chromosomes, while the single male with 51 chromosomes may represent a hybrid karyotype or a chromosomal aberration. Alternatively, an additional chromosome in males compared with females could, for example, also indicate an XX/XYY' sex determination system. The geographical spacing of populations with 50 and 52 chromosomes (Fig. 3) is contrasted by an increase of 10% in relative genome size along a west–east gradient, resulting in larger genomes in populations having fewer chromosomes. Assuming that the ancestral chromosome number was 52, an increase in relative genome size might be explained as a by-product of the fusion of two chromosomes. Alternatively, this gradient may represent an independent phenomenon, possibly linked to an ecological cline or varying retrotransposon activity.

We hypothesize that the change in chromosome number in *M. helleri* could have built up a reproductive barrier, thus initiating genetic divergence. The evolutionary implications differ from those in parthenogenetic species (e.g., *M. fuscistylis*), where asexuality itself is a barrier to gene flow. This becomes evident in our mitochondrial phylogeny, where karyotypically differing individuals did not form reciprocally monophyletic groups but showed some incongruence in the populations RAX and HWD (Fig. 3), possibly a consequence of limited, ongoing gene flow between the karyotypic groups. Recently, it has been shown that chromosomal rearrangements are key factors promoting speciation during secondary contact in several species pairs of rodents (Castiglia 2014). Similarly, the pattern seen in *M. helleri* could be interpreted as an early stage of speciation triggered by a chromosomal rearrangement.

#### Occurrence of B chromosomes

Varying numbers of B chromosomes were found in the sexual population of *M. ticinensis*. Interestingly, relative genome size significantly increased with the number of B chromosomes within this population, but the significant difference in relative genome size between sexual and parthenogenetic populations could not be explained by their presence alone. This is consistent with the results of Trivers et al. (2004), who found approx. 35% larger genomes in plants with reported B chromosomes compared with plants without B chromosomes. Because B chromosomes are thought to include a high amount of transposable elements (Camacho 2005), repeated transposition of mobile elements to the original chromosome set and subsequent proliferation could explain increased nuclear DNA content in the sexual population of *M. ticinensis*. Studying B chromosomes is an excellent opportunity to learn more about the dynamics of evolving genomes (Houben et al. 2014), and thus, *M. ticinensis* represents a prime study system for future research targeting the impact of B chromosomes on genome-size evolution.

## Data Accessibility

All DNA sequences were deposited at Genbank (accession numbers KJ501697 – KJ501918), including the final alignment used in this study (PopSet: 632799655). Individual chromosome counts and genome-size measurements are listed in Table S1 of the online supplemental material.

## Supporting Information

**Figure S1.** Karyotypes from selected individuals for each species included in this study

**Table S1.** Raw data and sampling information for all individuals included in this study.
