# Supplementary figures and images for: Extensive variation in chromosome number and genome size in sexual and parthenogenetic species of the jumping-bristletail genus Machilis (Archaeognatha)
Source: Ecol Evol. 2014 Oct 7;4(21):4093–105. doi: 10.1002/ece3.1264 (PMC4242562; doi:10.1002/ece3.1264)

**Fig. S1:** Karyotypes from selected individuals for each species included in this study.

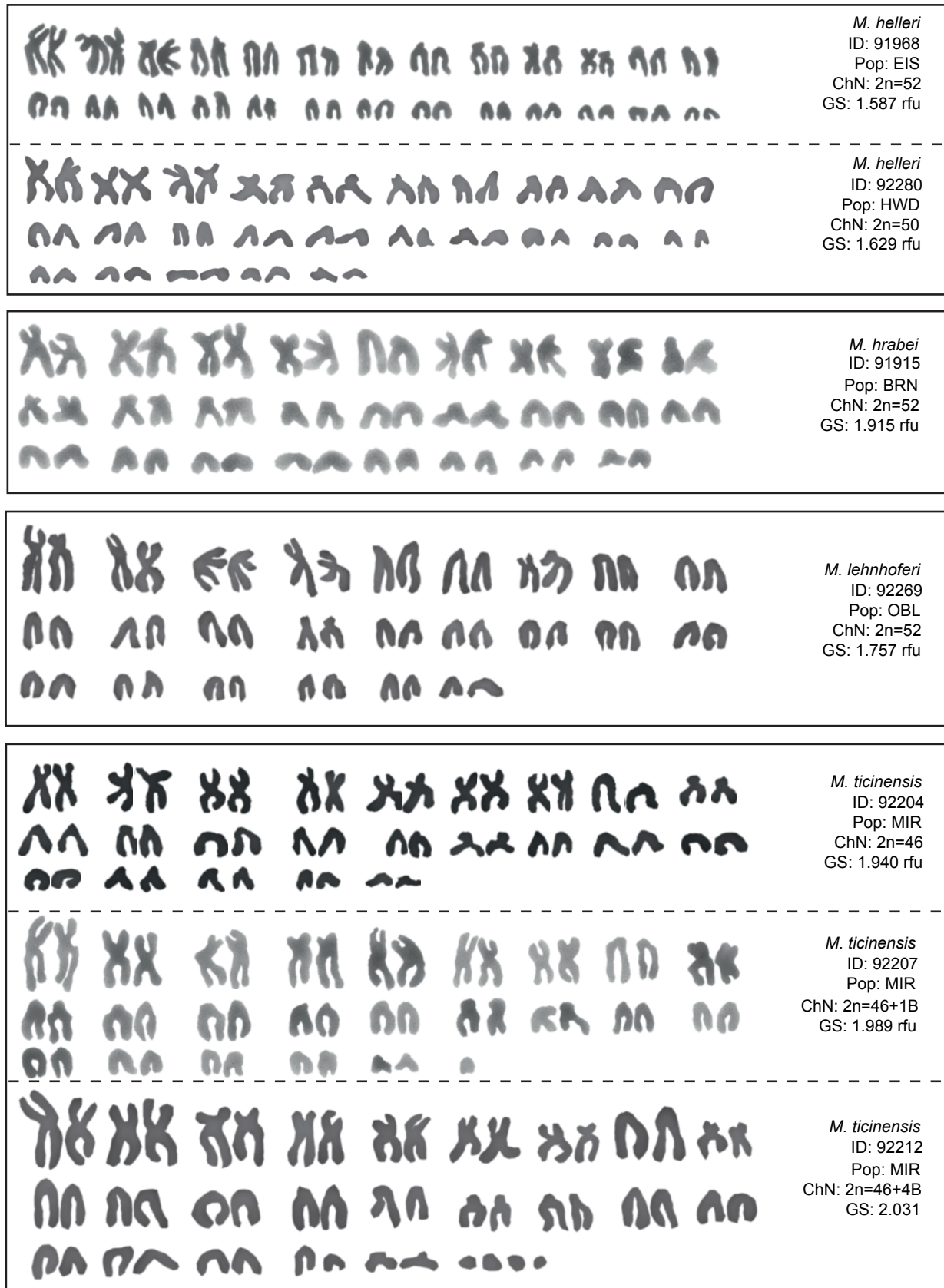

5  $\mu$ m

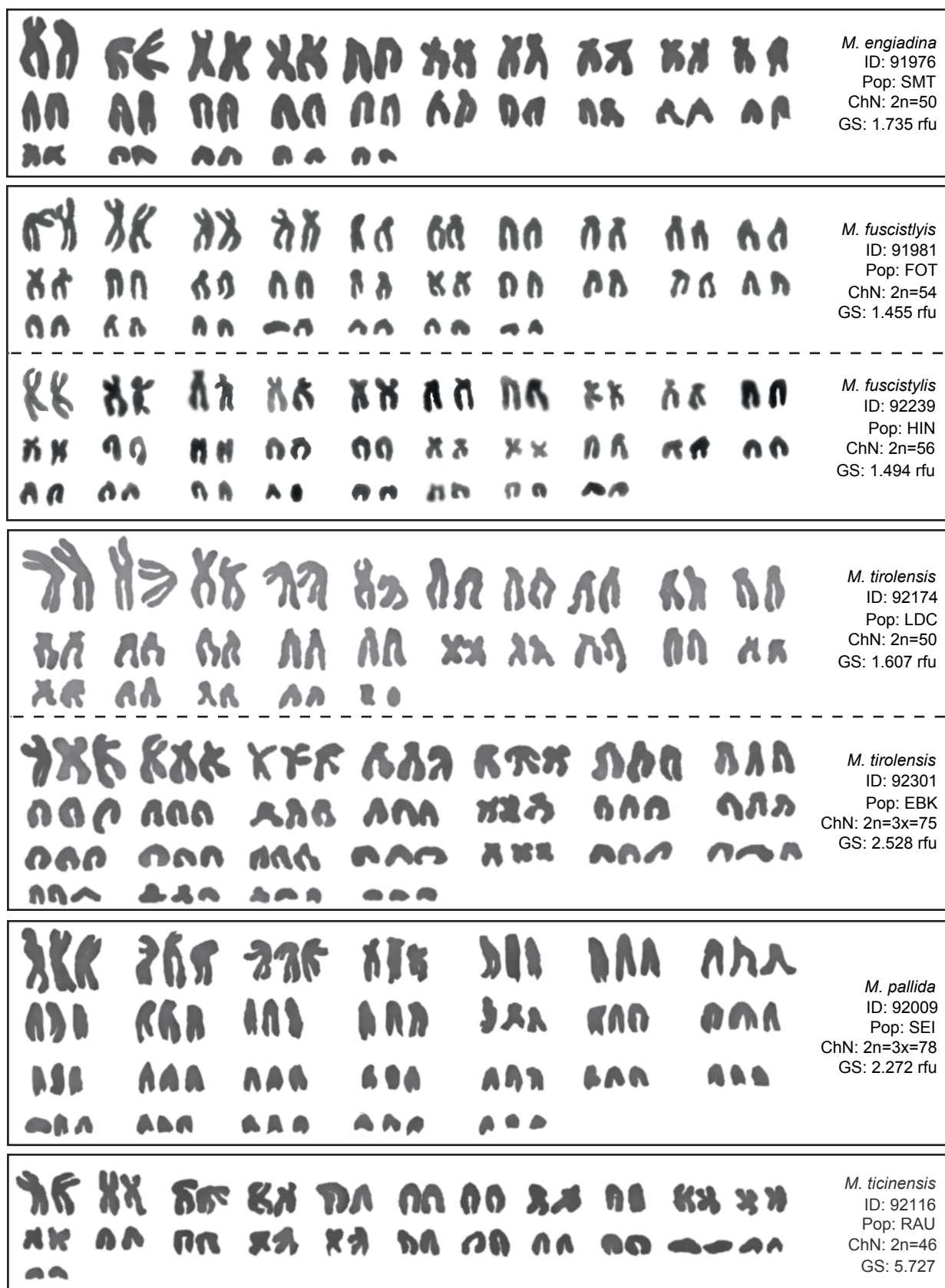

5  $\mu$ m

Supplement: Supplementary file 1 — Figure S1. Karyotypes from selected individuals for each species included in this study [file ece30004-4093-SD1.pdf]
